# Neuromyelitis optica presenting with relapses under treatment with natalizumab: a case report

**DOI:** 10.1186/1752-1947-8-155

**Published:** 2014-05-19

**Authors:** De-Hyung Lee, Alexandra B Laemmer, Anne Waschbisch, Tobias Struffert, Christian Maihöfner, Stefan Schwab, Ralf Andreas Linker

**Affiliations:** 1 Department of Neurology, Friedrich-Alexander University of Erlangen-Nuremberg, Schwabachanlage 6, 91054 Erlangen, Germany; 2 Department of Neuroradiology, Friedrich-Alexander University of Erlangen-Nuremberg, Schwabachanlage 6, 91054 Erlangen, Germany

**Keywords:** Neuromyelitis optica, Natalizumab, Multiple sclerosis, Rituxan, Anti-AQP-4 antibodies, Seroconversion

## Abstract

**Introduction:**

Neuromyelitis optica is an inflammatory demyelinating disease of the central nervous system. To date, optimal therapeutic approaches for neuromyelitis optica have yet to be defined. Natalizumab is highly effective in relapsing-remitting multiple sclerosis and might be considered as an option.

**Case presentation:**

Here, we describe a 67-year-old Caucasian man with definite neuromyelitis optica with detection of anti-aquaporin-4 antibodies over the course of the disease. After initially discussing the diagnosis of multiple sclerosis at an outside hospital, our patient received interferon beta 1a as well as repeated corticosteroid pulses without success. Under subsequent therapy with natalizumab, he continued to present relapses. It was not until discontinuation of natalizumab, repeated cycles of plasma exchanges and initiation of therapy with rituxan that the disease course started to stabilize. Although B cells were completely depleted, our patient experienced another severe myelitis relapse during further follow-up and an additional immunosuppressive therapy with cyclophosphamide was started. Under this regimen, no further relapses occurred over the next 24 months.

**Conclusions:**

This case adds further evidence to the previously discussed notion that natalizumab, while highly effective in multiple sclerosis, may not work sufficiently in neuromyelitis optica. It further advocates for repetitive testing of anti-aquaporin-4 antibodies before and after treatment initiation.

## Introduction

Neuromyelitis optica (NMO) is an immune-mediated demyelinating disease with predominant involvement of the optical nerve and the spinal cord. In contrast to multiple sclerosis (MS), NMO is associated with autoantibodies that target the aquaporin-4 water channel on astrocytes (anti-AQP-4 antibody). Anti-AQP-4 antibodies have both high specificity and sensitivity for NMO. AQP-4 seropositivity has been incorporated as additional criterion for the diagnosis of NMO [1,2]. For the treatment of NMO, small trials hint at positive effects of azathioprine, mitoxantrone, mycophenolate motefil and intravenous immunoglobulins, while several observations support the opinion that beta interferons have no effects or may even be harmful in NMO patients [3,4]. Nowadays, monoclonal antibodies targeting B cells such as rituxan play an increasingly important role in NMO therapy [5]. In addition, effects of further monoclonals like tocilizumab, eculizumab or natalizumab may be discussed [6,7].

## Case presentation

**Figure 1:**
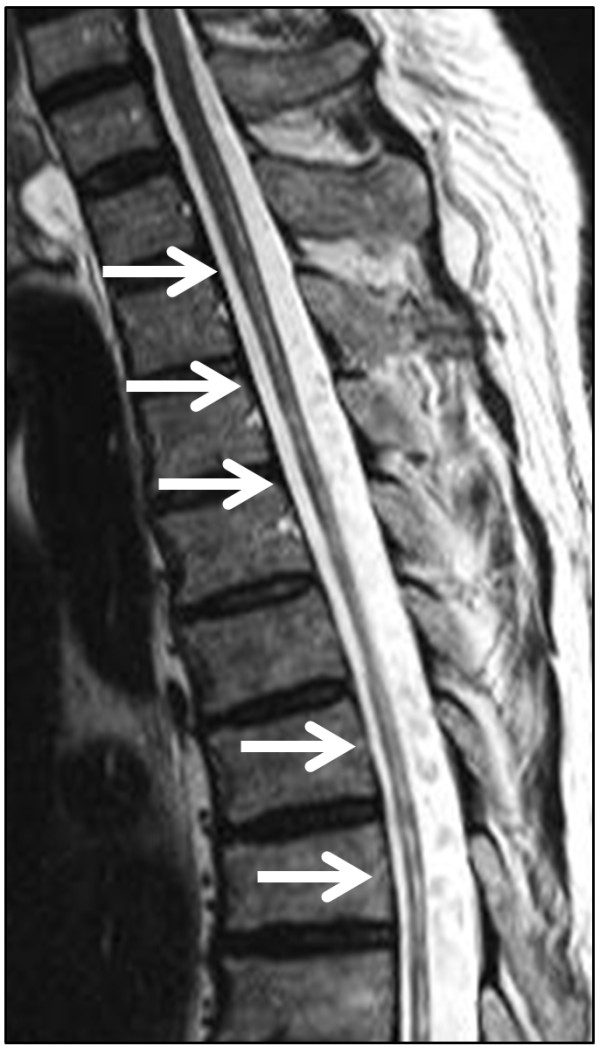
Spinal cord magnetic resonance imaging (T2-weighted imaging from C4 to Th6) one year after the start of combination treatment with rituxan and cyclophosphamide depicting longitudinally extensive spinal cord lesions (see arrows) with significant cord atrophy.

Without significant previous medical history, a 67-year-old Caucasian man developed spinal symptoms with temporary hypesthesia and hypoalgesia in both legs. These symptoms spontaneously resolved without any specific diagnosis at that time. At the age of 73, our patient suffered from bilateral optic neuritis and he was diagnosed with MS at an outside hospital. His expanded disability status scale (EDSS) score was at that time 2.5. Magnetic resonance imaging (MRI) studies of the spinal cord revealed a diffuse cord swelling and longitudinally extensive T2 hypertensive lesions extending from C2 to T3 (see Figure 1 depicting a T2-weighted MRI scan, which shows residual longitudinal myelitis with extensive cord atrophy). A cranial MRI scan displayed few periventricular and cerebellar lesions without contrast enhancement and without fulfilling the Barkhof criteria. Moreover, analysis of the cerebrospinal fluid (CSF) presented oligoclonal bands. At that time, anti-AQP-4 antibody testing was not performed. A therapy with interferon beta 1a was started for six months and was replaced by interferon beta 1b at the discretion of the treating outside neurologist. Our patient developed two further spinal relapses during the treatment with interferon beta preparations. They were treated with corticosteroid pulses without any success and his EDSS score worsened from 2.5 to 4.0. Although a subsequent therapy with natalizumab was initiated at an outside clinic, our patient continued to present another three relapses. The first relapse occurred four months after starting natalizumab, the second after six months and the third relapse after eight months. All relapses repeatedly affected both optic nerves and the spinal cord each with increasing visual and motor impairment. Thus, our patient developed a high-grade spastic tetraparesis as well as impaired visual acuity of both eyes and his EDSS score progressed from 4.0 to 8.0. At that point, NMO was discussed after referral to our hospital and natalizumab therapy was discontinued after nine courses. After repeated cycles of plasma exchange, the disease course stabilized and a therapy with rituxan was started. Although B cells were completely depleted, our patient experienced another severe myelitis relapse upon further follow-up three months later. Consequently, an additional immunosuppressive therapy with cyclophosphamide at a dosage of 600mg/m^2^ was initiated. In the meantime, we performed repetitive anti-AQP-4 antibody tests in an approved external laboratory employing an immunofluorescence assay (IFA) cell-based analysis. Negative anti-AQP-4 antibody tests were obtained via IFA analyses upon first admission and then in six-monthly intervals after first contact at our hospital. It was only after 18 months that anti-AQP-4 antibodies became positive after three negative results. At that time, the anti-AQP-4-immunoglobulin (Ig)G antibody titer was 1:1000 while IgM and IgA titers were negative. There were no other autoantibodies and no signs of other autoimmune diseases or malignancy.

Under combination treatment with cyclophosphamide (13 cycles every six weeks, cumulative dosage of 8300mg/m^2^) followed by another cycle of rituxan, our patient developed no further relapses over an observation period of 2.5 years.

## Conclusions

In this case NMO was initially misdiagnosed as MS, whereas CSF analysis revealed positive oligoclonal bands and an MRI scan of the brain presented some periventricular lesions without fulfilling the Barkhof criteria. However, oligoclonal bands may be positive in up to 30 percent of NMO patients and 60 percent of the patients may present supratentorial lesions [8]. In our patient, no follow-up CSF analysis was available to check for the persistence of oligoclonal bands.

After first- and second-line immunomodulatory therapy failed, natalizumab was chosen as an escalation therapy at an outside hospital. In at least one case series and two further case reports, a total of six NMO patients presented continuous relapses or even a deteriorating disease course under natalizumab, [6,9-11]. Subsequently, these observations were controversially discussed in the neuroimmunological community [12]. Our case adds further evidence to the notion that natalizumab, while highly effective in MS, may not work sufficiently in NMO. Indeed, natalizumab does not positively affect the trafficking properties of neutrophils, which are abundant in NMO lesions. After natalizumab therapy, an increase of B-cell precursors in the blood is observed, which might be counterproductive in case of a presumed B-cell-based pathomechanism as in NMO [13,14].

Our patient only stabilized after treatment with rituxan combined with cyclophosphamide. While cyclophosphamide alone may not work sufficiently in NMO [15], a combination treatment regime of complete B-cell depletion followed by cyclophosphamide pulses similar to rheumatologic disease may be more effective, at least in the present case. Nowadays, treatment with tocilizumab may be another option in rituxan-refractory NMO [7].

In our case, a first positive test for anti-AQP-4 antibodies occurred eight years after the onset of his first symptoms during a severe relapse. It is interesting to discuss whether this finding represents true seroconversion, is linked to variable test sensitivities or is governed by testing during a relapse. Indeed, titers of anti-AQP-4 antibodies might reflect the disease activity of NMO patients [16]. Thus, repeated testing in the active phases of the disease may prove useful. We thus propose critical testing for NMO in all patients with poorly controlled autoimmune central nervous system (CNS) inflammation, especially before, but also after initiation of natalizumab therapy. If NMO is clinically suspected but initial anti-AQP-4 antibody testing remains negative, repeated tests may be helpful to confirm the final diagnosis.

## Consent

Written informed consent was obtained from the patient for publication of this case report and any accompanying images. A copy of the written consent is available for review by the Editor-in-Chief of this journal.

## Abbreviations

AQP-4: aquaporin-4; CNS: central nervous system; CSF: cerebrospinal fluid; EDSS: expanded disability status scale; IFA: immunofluorescence assay; Ig: immunoglobulin; MRI: magnetic resonance imaging; MS: multiple sclerosis; NMO: neuromyelitis optica.

## Competing interests

The authors declare that they have no competing interests.

## Authors’ contributions

DL, AL and RL took the lead in drafting the manuscript and have made substantial contributions to interpretation of data. CM and SS have been involved in drafting the manuscript. AW and TS provided help with magnetic resonance imaging and critically revising the text. RL and CM revised it critically for important intellectual content. RL has given final approval of the version to be published. All authors read and approved the final manuscript.
